# Supplementary material for: Acceptance and compliance with micronutrient powder (MNP) among children aged 6–23 months in northern Nigeria
Source: PLOS Glob Public Health. 2022 Oct 17;2(10):e0000961. doi: 10.1371/journal.pgph.0000961 (PMC10022258; doi:10.1371/journal.pgph.0000961)
Supplement: S1 Dataset — (ZIP) [file pgph.0000961.s008.zip › S8_Dataset/In-depth Interviews.zip!/In-depth Interviews/110715_K_DW_TA_2_CG.a.pdf]

A PHASE TWO (2) HOME-FEEDING TRIAL IN-DEPTH INTERVIEW WITH A MALE CAREGIVER AT RUGA COMMUNITY, JEGA LOCAL GOVERNMENT AREA OF KEBBI STATE

ON 11TH JULY, 2015

CONDUCTED AND TRANSCRIBED BY TIMOTHY AUTA

I: Describe for me the people in your immediate family living in your household.

R: We are three in our family; my husband, me and the baby. My baby is just one year (twelve months). She is a girl.

I: We have heard of food challenges for young children in this setting related to the seasons.

R: The government should provide millet or sorghum that are needed for the preparation of kunu needed to mix the MNP with. The government can also help by ensuring that farmers get fertilizer on time to cultivate their crops so that we will have enough grains all seasons.

I: Ok. Thank you so much for that explanation. Could you please tell me how these challenges affect infant and young child feeding?

R: Some people don't have the food to use in feeding the child, especially in mixing this MNP. If this MNP is given to them, they will only keep it because they lack the food to use. This will affect the child tremendously.

I: Thanks. In our previews interviews, one thing we heard is that caregivers often give their children 6-23 months "tea". Please define or explain "tea".

R: We buy tea from the market. Tea is prepared by mixing hot water with milk, bournvita and sugar. One can also use Lipton in preparing tea.

I: Thanks for this explanation on tea. I Now, I want us to talk about the MNP that you received. Could you tell me about your experience with the product?

R: Well, my only experience is that the child was not eating food much before but since I started giving her the product, she has been eating more food.

I: Uh huh.

R: Silence.....

I: Please tell me more.

R: That's all I have to say.

I: OK. Please, talk about any positive aspects of the experience with MNP.

R: I am really impressed about it, the white colour is beautiful and the fact that it is given to the child to take is good.

I: What positive has it done to the child?

R: It has improved her health.

I: You just mentioned health. In what way did it improve the health?

R: Silence... She doesn't use to eat before but since she started taking it, she has started eating food.

I: Is that the only experience?

R: Yes, that is all the benefits I have seen.

I: Now, tell me about the baby before she starts taking the MNP and now that she is taking it.

R: What I have observed is that she is now stooling. She was not doing it before, but they told me is "dankanoma"

I: Uh huh

R: "Dankanoma" is the illness disturbing her.

I: So she was not stooling before?

R: Yes.

I: Could you please tell me more?

R: There is nothing.

I: Have you not observe any other thing since then?

R: She is now eating. Silence....

I: Ok. I want you to talk about any general challenges you had during the MNP trial.

R: The challenge is only one, the stooling. She has been stooling seriously.

I: When did she start this stooling?

R: About two weeks now.

I: What measure have you taken since then?

R: Silence.... It is medicines they say I will buy for him.

I: Have you bought the medicines?

R: No, I have not bought any.

I: Why?

R: Because I don't have money to buy the medicines.

I: But have you take her to the hospital?

R: No, I have not taken her to tthe hospital.

I: Ok. What specific barriers made it hard to use the MNP appropriately?

R: There is no barrier, it was not hard to use. It was very easy to use.

I. Some caregivers mentioned that it was hard to store the MNP without it being taken by others or eaten by rodents. Did you have any similar experience?

R: No, since I started storing it, no child or other person has touched it. I am the one that pick and give it to her. I don't send anyone to take it for me.

I: What are the perceived side effects of the MNP on the child?

R: It doesn't have any side effects.

I: Some mothers indicated that it might be hard to remember to use MNP. Did you have similar experiences?

R: No. Sincerely, I give her daily. What one was given to give his/her child free for the benefit of the child, why should one forgets? It is compulsory for one to always remember.

I: OK. What do you do that make you to always remember?

R: Now is fasting time. Whenever I prepare kunu, I take it and give it to her. This is what I do always.

I: Silence... Is that what makes you to remember?

R: Yes.

I: Tell me about more ways.

R: Silence...

I: Suggest specific solutions to address each of the above mentioned barriers or challenges.

R: For the rodents, it is rodenticides that should be used to kill them.

I: What about remembering to give?

R: The caregiver should store it where he/she can easily see to remember.

I: Thank you. Let's talk about the child's feeding and response to the MNP. Talk about how you feed the MNP to the child usually.

R: I use to give her with kunu. I fetch little of the kunu and mix it with the MNP. I don't give her very hot one, I give her warm kunu. I make sure I give her the one she can finish immediately. The next day, I do the same. That's what I do always.

I: Which food do you mix the MNP with?

R: We have already prepared "kumba" which we used during this fasting. That is what we use in preparing kunu that I use.

I: You mentioned "kumba", please describe it to me.

R: We dehusk millet and grind it to form the "kumba". It looks like the flour we use in preparing tuwo but we add spices to the millet before grinding when we want to prepare "kumba".

I: When do you usually give her the MNP?

R: In the morning.

I: Thank you. To what extent does the child likes or dislikes the taste of the MNP?

R: She doesn't like taking sometimes. I have to pamper her before she takes.

I: What is making her do dislike taking it?

R: She doesn't like taking kunu most times.

I: What does she take most?

R: She likes eating foods than gruel.

I: Ok. How did she find the taste, does she likes it?

R: She likes it. If I give her, she takes it.

I: OK. Earlier you mentioned that the baby eats more now, than before. Could you please me more on that?

R: Yes. She eats more now than she was doing before. She eats a lot of food now. She takes more breast milk now than before.

I: Thank you for that information. In other communities, we noticed that sharing of the MNP occurred. Could you tell me about any sharing when you used it?

R: No, she is the only one I give; I don't give any other person.

I: What about any sharing due to shortage of food during lean season?

R: She is the only one I have, so she eats her food alone. I don't allow her to eat with any other child within the neighbourhood. She eats alone.

I: Tell me about you and the father taking it as snack.

R: We don't take it. Since I collected it, I don't know how it tastes.

I: What happens to the leftover of the MNP mixed food?

R: I give her all to take. She takes everything.

I: Thanks so much. Will the 60 sachets be enough for 60 days?

R: When it finish, you may bring more.

I: When it is finished?

R: Yes.

I: But will the 60 sachets be enough for the 60 days?

R: 60 sachets for 60 days will be enough.

I: What makes you think it will be enough?

R: Because I give her one sachet per day. I have 60 sachets, that means it is to serve for the 60 days.

I: Now that you have used the MNP, could you talk about how you perceive it?

R: My perception is that it is good for all the children, so more should be distributed for all the children in the community to get.

I: Do you see the product as a food, a medicine or something else?

R: I see it as something that help the children.

I: What makes you think of it that way?

R: Because it helps children.

I: You just said it helps children. Would you purchase this product if it were cheaply on sale in your community?

R: I would buy.

I: What will make you buy?

R: If I have the money, because of the benefit I see in my daughter.

I: Silence...

R: Silence...

I: How much would you be willing to pay for each sachet?

R: If it is cheap, I will buy.

I: Like how much?

R: I can pay thirty naira (#30) on each sachet.

I: You're giving me great information. Now, please tell me some ways that you believe would be effective for promoting MNP to caregivers in the community so that they would comply with using it appropriately?

R: If you get people that will be going round from house to house, they will be checking to see how the people use it.

I: Uh huh.

R: Silence....

I: Please tell me more.

R: Silence....

I: Ok. What people most influence your opinions and knowledge about feeding practices?

R: Sometimes, my mother influences my opinions because she doesn't take feeding practices lightly.

I: Who influences your decisions regarding feeding your baby the MNP?

R: It is my mother that asked me to start giving her the MNP.

I: Where would be good places to disseminate health information related to MNP?

R: One can go the hospital for any health information.

I: What made you suggest hospital?

R: Because they are the ones that take care of people's health.

I: Ok. Who in your community, do you trust the most for receiving messages about infant and young child feeding?

R: The community leader, I trust him and believe he won't do anything that will affect us negatively.

I: Ok. What forms of media do you prefer for receiving MNP messages? Poster/Brochure/Flyer?

R: Yes, they are good to people that can read. But since we cannot read,

we will need someone to explain them to us.

I: What about word-of-mouth?

R: It will be good. Because one can easily ask any question about what he/she doesn't understand or need more information on.

I: From whom would you want to?

R: From the community leader. If you explain to him, he can also explain to us.

I: What about radio?

R: Yes, radio will be good. Because through the radio, we can get more information and explanations

I: What about SMS?

R: That one is for those who can read and have handset. For me, I don't have handset. I will prefer word-of-mouth.

I: Is there any other form of media you think should be used?

R: This can be done through radio, handsets and through discussions like this.

I: Tell me preferred ways of receiving education regarding IYCF and MNP.

R: Through the health workers. They are the right people through whom education or any information can be rightly disseminated to the people.

I: Give me specific examples of other successful health education campaigns in this community.

R: I don't know of any such campaigns in this community.

I: What are the best channels in this community to distribute the product?

R: It should be done through the community leader's palace. If you bring it there, you can give it to workers who will go from house-to-house distributing, just as is done during polio immunization. They should enter houses to give caregivers in their hands.

I: Why do you think of that channel?

R: Because that's what I see the polio immunization doing. During immunization, they go from house-to-house.

I: Thank you for that suggestion. I want to hear some specific messages you think would effectively promote the product.

R: I don’t have any message for that.

I: What messages should be used to explain MNP to caregivers?

R: They should be told the MNP is good because it gives the child good health.

I: Uh huh

R: It also makes the child to eat if he/she was not eating well before.

I: Uh huh.

R: (Silence...)

I: Tel me more messages.

R: That's all I have to say.

I: OK. What specific messages will ensure appropriate use of the MNP?

R: Those who want their children to be healthy don't ignore the product. They will always ensure they give it to their children. They don't joke with it because of its importance. If they see others taking out of it, they won't allow him/her.

I: OK. What messages will be effective to promote its use but limit the sharing of it.

R: When you come, you check to ensure they are using it. Count the number they have left from the one you gave them, if they have been using it, you will know and if they have not been using it, you will also know.

I: I mean what messages should be given to them.

R: They should not share it with others because it will make it not to work as expected. When preparing to give to the child, they should only mix it with the amount of food the child can finish immediately, there should not be leftover.

I: OK. Finally, is there anything else that you can share about your experience with this product?

R: What I have to say is I am grateful for this MNP and plead with the government to please try and bring more for the other children to benefit from.

I: What else can you recommend to develop an effective program?

R: My recommendation is that the government should help with food materials that can be used to feed the child with the MNP.

I: Thanks so much for your time.
